# Supplementary material for: Machine learning assistive rapid, label-free molecular phenotyping of blood with two-dimensional NMR correlational spectroscopy
Source: Commun Biol. 2020 Sep 28;3:535. doi: 10.1038/s42003-020-01262-z (PMC7522972; doi:10.1038/s42003-020-01262-z)
Supplement: Supplementary file 1 — Supplementary Information [file 42003_2020_1262_MOESM1_ESM.pdf]

Machine learning assisted rapid, label-free molecular phenotyping of blood with two-dimensional NMR correlational spectroscopy

Weng Kung Peng[1]*, Tian-Tsong Ng[2], Tze Ping Loh[3]*

[1]Precision Medicine – Engineering Group, International Iberian Nanotechnology Laboratory, 4715 330 Braga - Portugal
[2]Institute for Infocomm Research, Fusionopolis Way, Singapore
[3]Department of Laboratory Medicine, National University Hospital, Singapore

Corresponding author: W. K. Peng (weng.kung@inl.int), T. P. Loh (tze_ping_loh@nuhs.edu.sg)

Supplementary Figures.

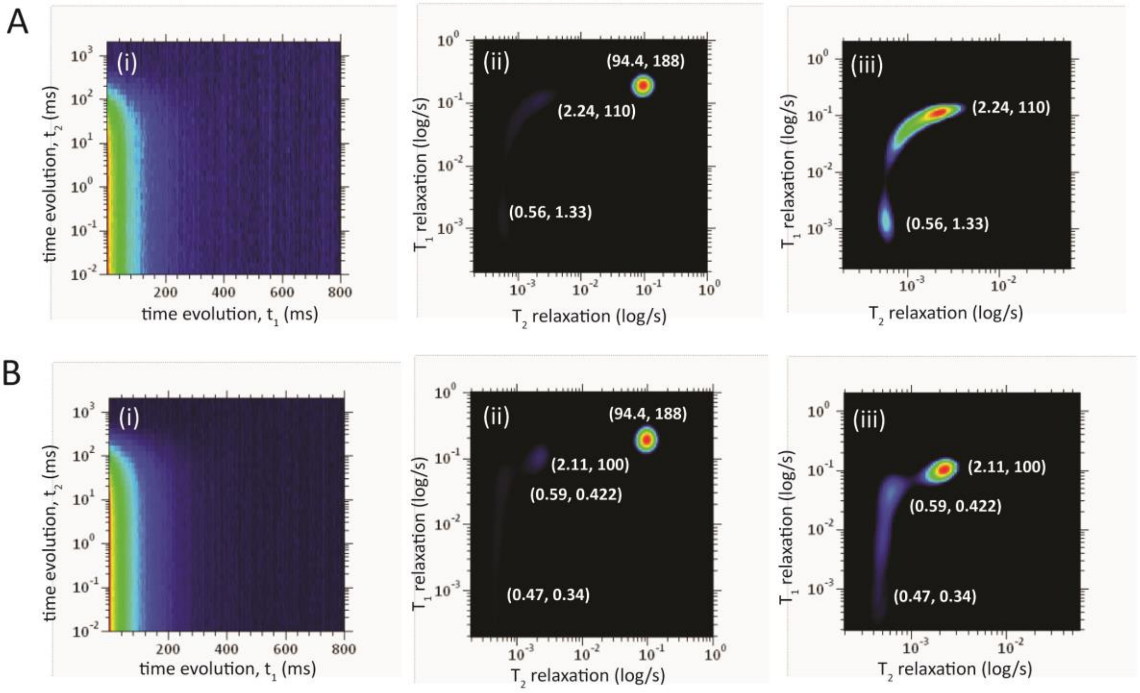

Supplementary Figure 1. Rapid signl acquisition and short spectra inversion time with (i) raw echoes signal, (ii) Laplace inversion correlational map, and (iii) zoom-in details of fast relaxation components (S-peak and T-peak). The echo time used were 200 µs, number of echoes were 4000 echoes, and T_1-incremental were 40 (logarithmic) steps and the signal averaging were (a) 4 scans, and (b) 16 scans. The total experiment times were (a) 6 minutes, and (b) 24 minutes, respectively. The optimal SNR obtained is approximately 1600.

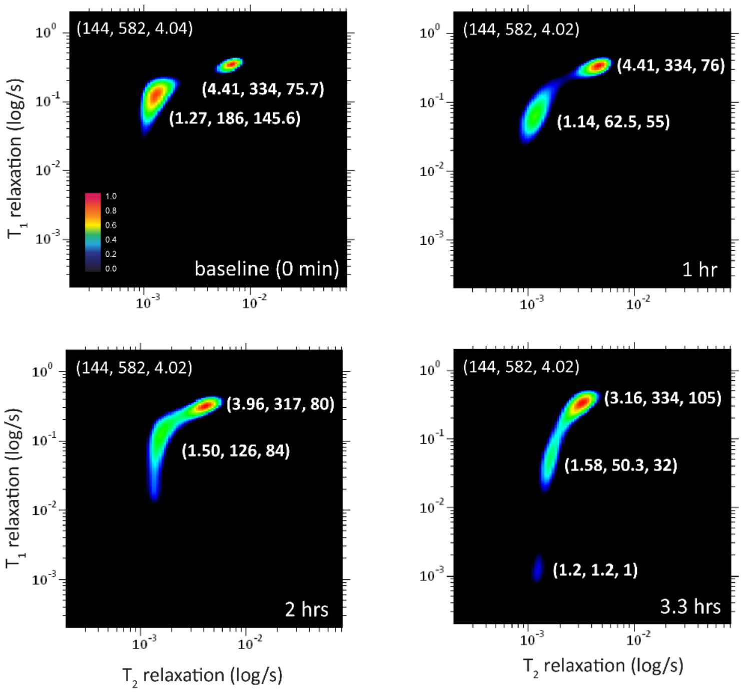

Supplementary Figure 2. Oxidative degradation of hemoglobin in *ex-vivo* condition. The zoom-in details of decomposed relaxation reservoirs for fast relaxation components (S-peak and T-peak), while the slow relaxation component (bulk water molecules, R-peak) is not shown. The coordinate for R-peak is indicated at upper left of the spectrum. The gradual degradation of hemoglobin correspond to the gradual increase of blood oxidation activities (met-Hb concentration). The micro MR measurements were carried out periodically at time-point as indicated above. Control baseline is indicated as '0 min' with hemoglobin in predominantly the oxygenated state. The coordinate is represented as (T_2 relaxation (in ms), T_1 relaxation (in ms), A-ratio (arb)).

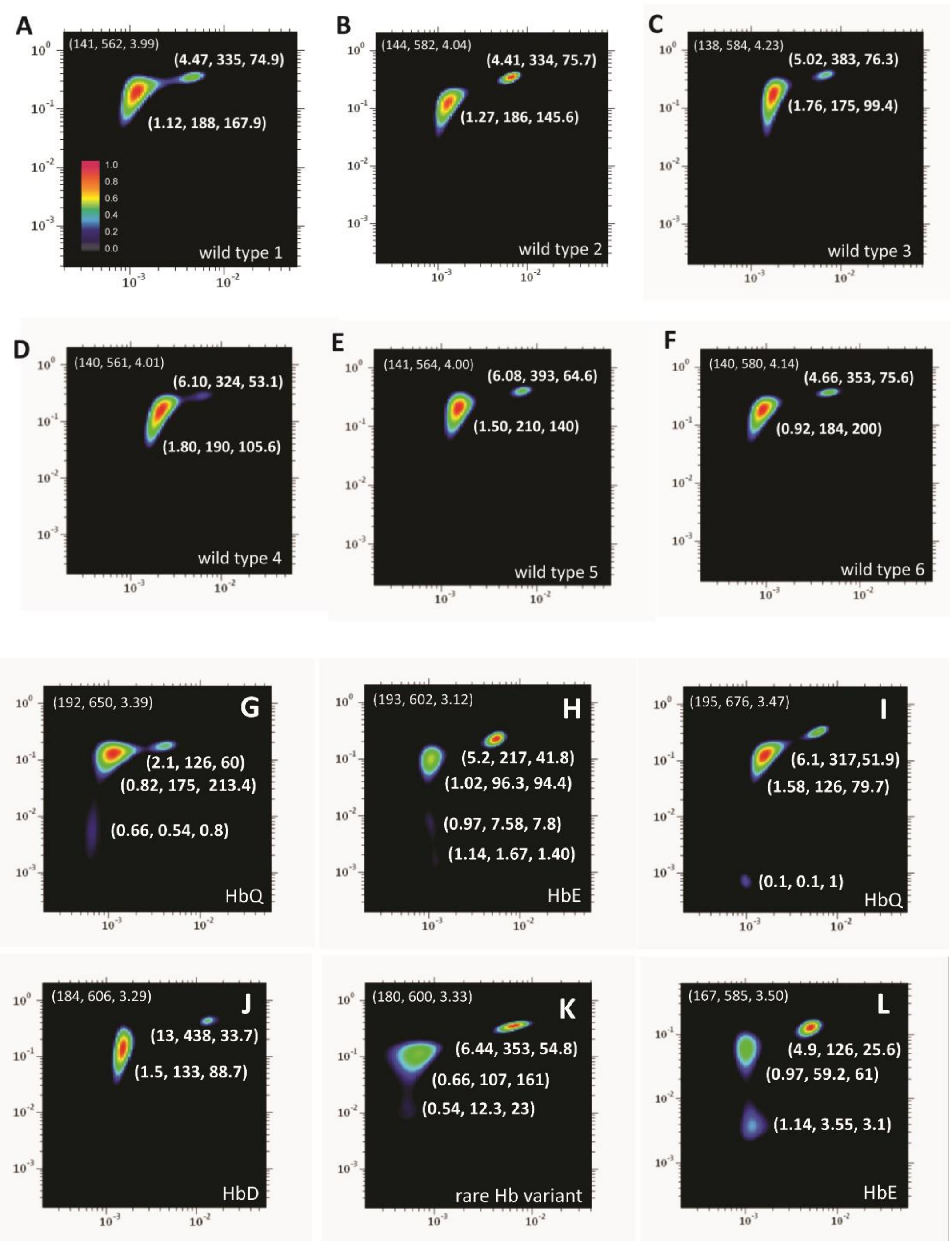

Supplementary Figure 3. (a) The T_1-T_2 correlational spectrum of blood microenvironment of healthy normal hemoglobin (control) taken from six healthy donors (A to F), denoted as wild type 1 to wild type 6, and (b) Hb variants (G to L). The zoom-in details of decomposed relaxation reservoirs for fast relaxation components (S-peak and T-peak), while the slow relaxation component (bulk water molecules, R-peak) is not shown. The coordinate for R-peak is indicated at upper left of the spectrum. The details of the coordinate is summarized in Supplementary Table 2.

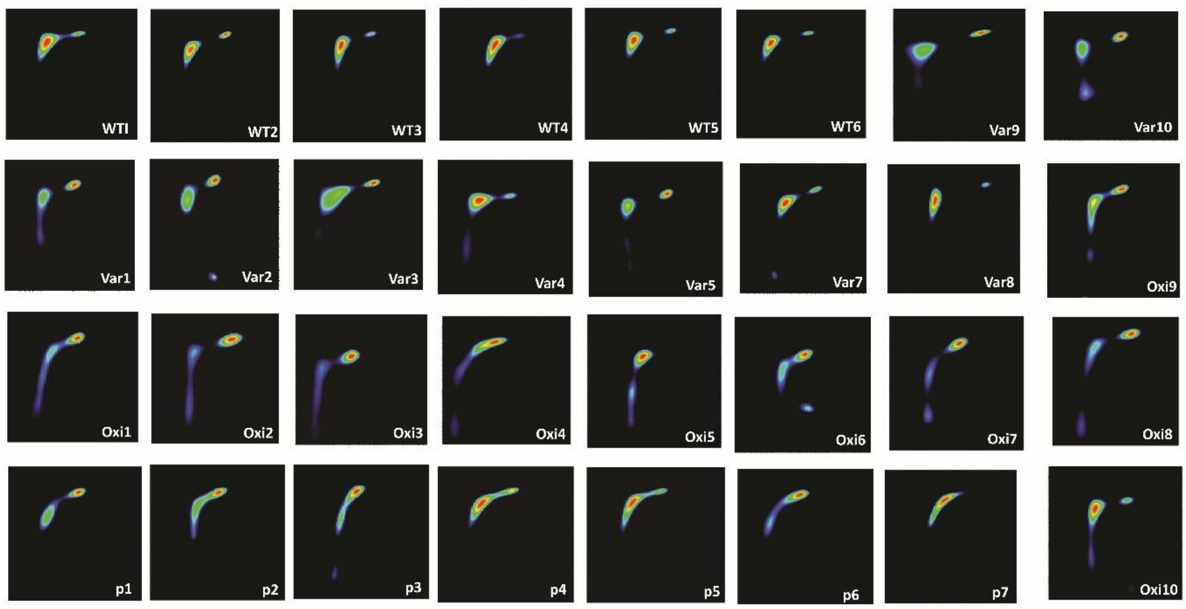

Supplementary Figure 4. The T_1-T_2 correlational spectrum of blood microenvironment of 32 anonymized subjets used for disease clustering and to evaluate blind test performance.

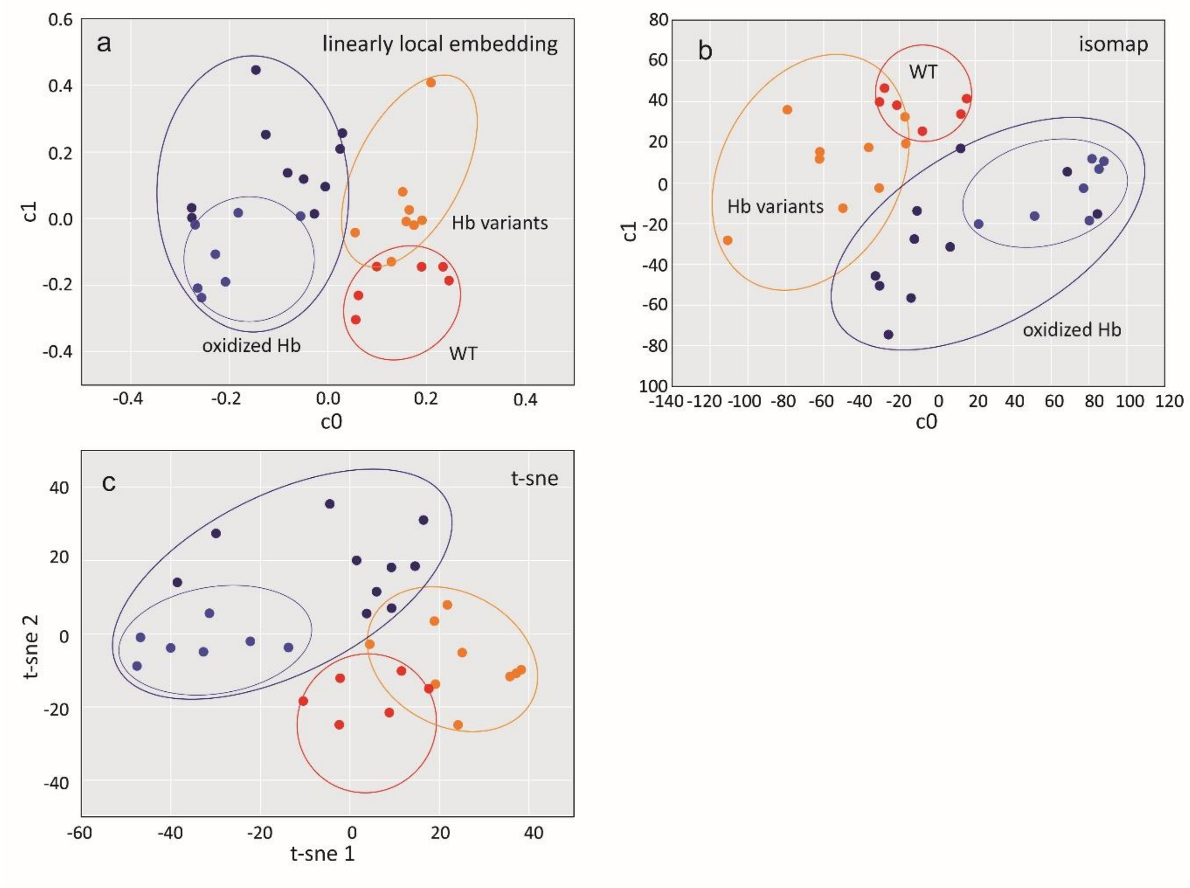

Supplementary Figure 5. Classification of two states (disease, non-disease, Hb variants) and disease subtyping (sub-type 1: oxidized Hb, sub-type 2: partially oxidized Hb) using (a) locally linear embedding technique (method: local, 25 neighbors, 200 max iterations), (b) Isomap technique (10 neighbors, 2 outputs components), (c) t-sne technique (60 perplexity, 12 early exaggeration, 200 learning rate, max iterations, 4 outputs components).

image	Label	Category	Obs 1 (M)	Obs 2 (J)	Obs 3 (M)	Obs 4 (Mn)	Obs 5 (T)
1	WT1	healthy	healthy	healthy	healthy	healthy	healthy
2	WT2	healthy	disease	healthy	healthy	healthy	healthy
3	WT3	healthy	healthy	healthy	healthy	healthy	healthy
4	WT4	healthy	healthy	healthy	disease	disease	healthy
5	WT5	healthy	healthy	healthy	healthy	disease	disease
6	WT6	healthy	healthy	disease	disease	disease	healthy
7	Hb Var 1	disease	disease	disease	disease	disease	disease
8	Hb Var 2	disease	healthy	healthy	disease	disease	disease
9	Hb Var 3	disease	disease	disease	disease	disease	disease
10	Hb Var 4	disease	disease	disease	disease	disease	disease
11	Hb Var 5	disease	disease	disease	disease	disease	disease
12	Hb Var 6	disease	disease	disease	disease	disease	disease
13	Hb Var 7	disease	disease	disease	disease	disease	disease
14	Hb Var 8	disease	healthy	healthy	healthy	healthy	healthy
15	Hb Var 9	disease	disease	disease	disease	disease	disease
16	Oxi Hb	disease	disease	disease	disease	disease	disease
17	Oxi Hb	disease	disease	disease	disease	disease	disease
18	Oxi Hb	disease	disease	disease	disease	disease	disease
19	Oxi Hb	disease	disease	healthy	disease	disease	disease
20	Oxi Hb	disease	disease	disease	disease	disease	disease
21	Oxi Hb	disease	disease	disease	disease	disease	healthy
22	Oxi Hb	disease	healthy	healthy	healthy	disease	healthy
23	Oxi Hb	disease	healthy	healthy	disease	healthy	disease
24	Oxi Hb	disease	disease	disease	disease	disease	disease
25	Oxi Hb	disease	disease	disease	disease	disease	disease
26	Oxi Hb	disease	disease	disease	disease	disease	disease
27	Oxi Hb	disease	disease	disease	disease	disease	disease
28	Oxi Hb	disease	disease	disease	disease	disease	disease
29	Oxi Hb	disease	healthy	healthy	disease	healthy	healthy
30	Oxi Hb	disease	healthy	healthy	healthy	healthy	healthy
31	Oxi Hb	disease	disease	disease	disease	disease	disease
32	Oxi Hb	disease	disease	disease	disease	disease	disease

Supplementary Figure 6a. The details of the blind test; (a) human classification and (b) performance. The terminology used were True Positive (TP), True Negative (TN), False Positive (FP), and False Negative (FN).

	image	Obs 1 (M)	Obs 2 (J)	Obs 3 (M)	Obs 4 (Mn)	Obs (T)
	1	TN	TN	TN	TN	TN
	2	FP	TN	TN	TN	TN
	3	TN	TN	TN	TN	TN
	4	TN	TN	FP	FP	TN
	5	TN	TN	TN	FP	FP
	6	TP	FP	FP	FP	TP
	7	TP	TP	TP	TP	TP
	8	FN	FN	TP	TP	TP
	9	TP	TP	TP	TP	TP
	10	TP	TP	TP	TP	TP
	11	TP	TP	TP	TP	TP
	12	TP	TP	TP	TP	TP
	13	TP	TP	TP	TP	TP
	14	FN	FN	FN	FN	FN
	15	TP	TP	TP	TP	TP
	16	TP	TP	TP	TP	TP
	17	TP	TP	TP	TP	TP
	18	TP	TP	TP	TP	TP
	19	TP	FN	TP	TP	TP
	20	TP	TP	TP	TP	TP
	21	TP	TP	TP	TP	FN
	22	FN	FN	FN	TP	FN
	23	FN	FN	TP	FN	TP
	24	TP	TP	TP	TP	TP
	25	TP	TP	TP	TP	TP
	26	TP	TP	TP	TP	TP
	27	TP	TP	TP	TP	TP
	28	TP	TP	TP	TP	TP
	29	FN	FN	FN	TP	FN
	30	FN	FN	FN	FN	FN
	31	TP	TP	TP	TP	TP
	32	TP	TP	TP	TP	TP
	TP	21	19	22	23	22
	FP	1	1	2	3	1
	TN	4	5	4	3	4
	FN	6	7	4	3	5
	Total	32	32	32	32	32
Sensitivity	TP / (TP + FN)	0.78	0.73	0.85	0.88	0.81
Specificity	TN / (FP + TN)	0.80	0.83	0.67	0.50	0.80
Precision	TP / (TP + FP)	0.95	0.95	0.92	0.88	0.96
F1 score	2TP / (2TP + FN + FP)	0.86	0.83	0.88	0.88	0.88
ACC	(TP + TN) / (all)	0.78	0.75	0.81	0.81	0.81

Supplementary Figure 6b. The details of the blind test. The terminology used were True Positive (TP), True Negative (TN), False Positive (FP), and False Negative (FN).

k=2	AUC	CA	Sensitivity	Specificity	Precision	F1
neural network	0.987	0.812	0.812	1	0.906	0.832
kNN	0.885	0.875	0.875	0.67	0.875	0.875
logistic regression	0.897	0.812	0.812	0.5	0.812	0.812
naïve bayes	0.5	0.188	0.188	1	0.035	0.059
average	0.817	0.672	0.672	0.793	0.657	0.645

k=3	AUC	CA	Sensitivity	Specificity	Precision	F1
neural network	0.958	0.844	0.875	1	0.925	0.885
kNN	0.896	0.812	0.812	0.67	0.839	0.822
logistic regression	0.963	0.906	0.906	0.83	0.914	0.909
naïve bayes	0.595	0.344	0.344	1	0.854	0.33
average	0.853	0.727	0.734	0.874	0.883	0.737

k=5	AUC	CA	Sensitivity	Specificity	Precision	F1
neural network	0.92	0.906	0.906	1	0.938	0.913
kNN	0.912	0.844	0.844	0.83	0.881	0.855
logistic regression	0.927	0.906	0.906	0.83	0.914	0.909
naïve bayes	0.697	0.406	0.406	1	0.858	0.417
average	0.864	0.766	0.766	0.915	0.898	0.774

leave one-out method	AUC	CA	Sensitivity	Specificity	Precision	F1
neural network	0.994	0.875	0.875	1	0.925	0.885
kNN	0.936	0.875	0.875	1	0.925	0.885
logistic regression	0.917	0.906	0.906	0.83	0.914	0.909
naïve bayes	0.788	0.562	0.562	1	0.869	0.6
average	0.909	0.805	0.805	0.958	0.908	0.820

Supplementary Figure 7. The details of the supervised learning performance using various k-fold cross validation sampling (e.g., k=2, k=3, and k=5), and leave-one-out methods, respectively. The performance of the naïve Bayes model was found to be unsuitable for this study, if the k-fold sampling is small (e.g., k<5).

Supplementary Table

reference	immuno-labelling	disease	POCT	AI-assisted	Time to results	info
this work	label-free	hemoglobinopathies	Yes	Yes	min	2D
Peng [7–10]	label-free	malaria screening	Yes	No	min	1D
Lee[11]	label-based	bacteria detection	Yes	No	min to hour	1D
Haun[12]	label-based	cancer (breast)	Yes	No	min to hour	1D
Haun[13]	label-based	cancer (intra-abdominal)	Yes	No	min to hour	1D
Liong[14]	label-based	M. Tuberculosis	Yes	No	min to hour	1D
Chung[15]	label-based	bacteria detection	Yes	No	min to hour	1D
Gee[16]	label-based	cancer (melanoma)	Yes	No	min to hour	1D
Lei[17]	label-based	bacteria detection	Yes	No	min to hour	1D
Ha[18]	not related	no information	Yes	No	min	2D

Supplementary Table 1. Summary of recently developed NMR-based POCT technologies which uses time-domain signal (or known as relaxometry), and its´ salient features. The terminology used were min = minutes, hr = hour.

	relaxation time (ms)			*(P-value)*	
Bulk water, R-peak	T_1 (ms)	T_2 (ms)	A ratio	T_1 (ms)	T_2 (ms)
Hb wild type (control)	562	141	3.99	0.0004	0.00004
Hb wild type (control)	582	144	4.04	P<0.0005	P<0.0005
Hb wild type (control)	584	138	4.23		
Hb wild type (control)	561	140	4.01		
Hb wild type (control)	564	141	4.00		
Hb wild type (control)	580	140	4.14		
HbE variant	631	158	3.99		
HbD variant	640	165	3.88		
rare Hb variant	640	165	3.88		
HbE variant	602	193	3.12		
HbE variant	585	167	3.50		
rare Hb variant	600	180	3.33		
HbD variant	606	184	3.29		
HbQ	676	195	3.47		
HbQ	650	192	3.39		

	relaxation time (ms)			*(P-value)*	
Hydration layer, S-peak	T_1 (ms)	T_2 (ms)	A ratio	T_1 (ms)	T_2 (ms)
Hb wild type (control)	335	4.47	74.94	0.16	0.49
Hb wild type (control)	334	4.41	75.74	P<0.5	P<0.5
Hb wild type (control)	383	5.02	76.29		
Hb wild type (control)	324	6.10	53.11		
Hb wild type (control)	393	6.08	64.64		
Hb wild type (control)	353	4.66	75.75		
HbE variant	335	4.42	75.79		
HbD variant	373	4.18	89.23		
rare Hb variant	362	6.48	55.86		
HbE variant	217	5.2	41.73		
HbE variant	126	4.9	25.71		
rare Hb variant	353	6.44	54.81		
HbD variant	438	13	33.69		
HbQ	317	6.1	51.97		
HbQ	126	2.1	60.00		

	relaxation time (ms)			*(P-value)*	
Direct bound, T-peak	T_1 (ms)	T_2 (ms)	A ratio	T_1 (ms)	T_2 (ms)
Hb wild type (control)	188.00	1.12	167.9	0.0004	0.17
Hb wild type (control)	186.00	1.27	146.5	P<0.0005	P~0.15
Hb wild type (control)	175.00	1.76	99.4		
Hb wild type (control)	190.00	1.80	105.6		
Hb wild type (control)	210	1.5	140.0		
Hb wild type (control)	184	0.92	200.0		
HbE variant	106	1.06	100.0		
HbD variant	96.3	1.20	80.3		
rare Hb variant	172	1.40	122.9		
HbE variant	96.3	1.02	94.4		
HbE variant	59.2	0.97	61.0		
rare Hb variant	107	0.66	162.1		
HbD variant	133	1.5	88.7		
HbQ	126	1.58	79.7		
HbQ	175	0.82	213.4		

Supplementary Table 2. The summary of T_1 relaxations and T_2 relaxations (for R-peak, S-peak and T-peak) of the healthy normal hemoglobin (wild-tpye) as compared to other hemoglobin variants. The (R-peaks, T-peaks) were statistically significant (P<0.05), while S-peaks were not statistically significant from the controls (wild type). The two-tailed Student´s T-test was used to calculate the *P*-value.

Supplementary Discussion.

Rapid signal acquisition and spectral inversion time. One of the biggest challenge in implementation of multidimensional inverse Laplace correlational spectroscopy is the undesirable long experiment time (e.g., signal acquisition time) and long post processing time (e.g., Laplace inversion time)[1,2]. The long signal acquisition is inherently due to the long dephasing time (typically a few seconds for liquid NMR) and low signal to noise ratio (SNR).

In this work, however, we used red blood cells (RBCs) which has semi solid structure as our target sample and the proton nuclei of RBCs under observation had relatively short transverse relaxation time (less than 200 ms) shorter than free water (2.5 sec). For each acquisition, more than 20 folds of experimental time has been shortened, and for an entire 2D NMR expriement (with e.g., 24 acquisition), more than 400 folds of experimental time has been shortened.

In order to evaluate the quality of the raw echoes signal acquired and the Laplace inversion correlational map, we carried out two succsesive acquistions on freshly prepared met-Hb (refer to Methods Online), using (a) 4 scans and (b) 16 scans (Supplementary Fig. 1) as signal averaging. The total acquistion time taken were 6 minutes and 24 minutes, for 4 and 16 scans, respectively. The SNR would improve but at the expenses of acquisition time. The 2D correlation maps were processed using built-in ILT algorithm (FISTA inversion[3], KEA built in-function) method with 5000 iterations, and smoothing parameter of 1 were used to produce a 200 x 200 steps correlational map. The inversion typically completed in less than 1 minute using a desktop computer (Intel Core Pentium i3 CPU @ 3.2GHz, 1.74Gb RAM).

We observed that the bulk water molecules main peaks and two other short relaxation peaks (sub-ms) were reproduced in both cases. Furthermore, the bulk water molecules peaks coordinate were (T_2=94.4ms, T_1=188ms) retained (Supplementary Fig. 1A (ii) - 1B (ii)). As for short relaxation peaks (sub-ms), there were minor shift (Supplementary Fig. 1A (iii) - 1B (iii)), as the experiments were performed sequentially. The minor shift is to be expected as the cells' biologic evolved over time, in particularly in the case of hemoglobin oxidation. Essentially, important feature were clearly retained in these two successive acquisition. The RBCs sample used in this work has much shorter dephasing time (less than 200ms), and with the 4 signal averaging scans (Supplementary Fig. 1a), sufficiently high SNR was recorded in less than 6 minutes of total experiment time. The Laplace inversion time completes in less than 2 minutes.

In conclusion, it is demonstrated that the combined signal acquisition and Laplace inversion time shortened to minutes, thus paving way for the possibility of rapid molecular phenotyping at point-of-care setting. In addition, with the recent availability of ultrafast signal acquisition methods[2,4] and efficient inversion algortihm[1,5], real-time characterization and monitoring may be feasible[6].

Oxidative degradation of hemoglobin. The whole blood were exposed to mild oxidant of 0.01 mM of sodium nitrite were exposed for 10 minutes and the reaction were quenched by washing the sample three times with PBS, resuspend in PBS, and spun down (6000*g*, 1 min) using a microcapillary tubes meant for NMR measurements. The 2D correlational spectrum were taken sequentially a over period of 3.3 hours to observe the hemoglobin degradation process (Supplementary Fig. 2). The fast relaxation components (T-peak) dropped to (T_2=1.14ms, T_1=62.5ms) from its' original state (T_2=1.27ms, T_1=186ms), indicating that a significant amount

of oxidation had taken place over a period of one hour. A small sub-population of the red blood cells continued to be oxidized, causing the formation of T_1-relaxation stretching (2 hrs), which accumulated continuously (3.3 hrs). A subpopulation of the hemoglobin, which were most likely due to the aged red blood cells with depleted anti-oxidant capacity, continued to be locked in the oxidized state, forming the signature T_1 relaxation stretching ending at peak (T_2=1.2ms, T_1=1.2ms) 3.3 hrs. It is worth noting that the process of oxidation and reduction is a dynamic process, and this process is mediated by the presence of anti oxidant (e.g., gluthathione) which act as buffer for recovery process. This is an unique and personalized features which can be exploited for personalized medicine (e.g., drug screening).

References

1. Song, Y.-Q. *et al.* T1–T2 Correlation Spectra Obtained Using a Fast Two-Dimensional Laplace Inversion. *J. Magn. Reson.* **154**, 261–268 (2002).
2. Ahola, S. & Telkki, V.-V. Ultrafast Two-Dimensional NMR Relaxometry for Investigating Molecular Processes in Real Time. *ChemPhysChem* **15**, 1687–1692 (2014).
3. Zhou, X., Su, G., Wang, L., Nie, S. & Ge, X. The inversion of 2D NMR relaxometry data using L1 regularization. *J. Magn. Reson.* **275**, 46–54 (2017).
4. Ahola, S. *et al.* Ultrafast multidimensional Laplace NMR for a rapid and sensitive chemical analysis. *Nat. Commun.* **6**, (2015).
5. Song, Y.-Q., Ryu, S. & Sen, P. N. Determining multiple length scales in rocks. *Nature* **406**, 178–181 (2000).
6. Peng, W. K. & Paesani, D. Omics Meeting Onics: Towards the Next Generation of Spectroscopic-Based Technologies in Personalized Medicine. *J. Pers. Med.* **9**, 39 (2019).
7. Peng, W. K. *et al.* Micromagnetic resonance relaxometry for rapid label-free malaria diagnosis. *Nat. Med.* **20**, 1069–1073 (2014).
8. Han, J. & Peng, W. K. Reply to 'Considerations regarding the micromagnetic resonance relaxometry technique for rapid label-free malaria diagnosis'. *Nat. Med.* **21**, 1387–1389 (2015).
9. Dupré, A., Lei, K.-M., Mak, P.-I., Martins, R. P. & Peng, W. K. Micro- and nanofabrication NMR technologies for point-of-care medical applications – A review. *Microelectron. Eng.* **209**, 66–74 (2019).
10. Veiga, M. I. & Peng, W. K. Rapid phenotyping towards personalized malaria medicine. *Malar. J.* **19**, 68 (2020).
11. Lee, H., Sun, E., Ham, D. & Weissleder, R. Chip–NMR biosensor for detection and molecular analysis of cells. *Nat. Med.* **14**, 869–874 (2008).
12. Haun, J. B., Devaraj, N. K., Hilderbrand, S. A., Lee, H. & Weissleder, R. Bioorthogonal chemistry amplifies nanoparticle binding and enhances the sensitivity of cell detection. *Nat. Nanotechnol.* **5**, 660–665 (2010).
13. Haun, J. B. *et al.* Micro-NMR for Rapid Molecular Analysis of Human Tumor Samples. *Sci. Transl. Med.* **3**, 71ra16-71ra16 (2011).
14. Liong, M. *et al.* Magnetic barcode assay for genetic detection of pathogens. *Nat. Commun.* **4**, 55–65 (2013).
15. Chung, H. J., Castro, C. M., Im, H., Lee, H. & Weissleder, R. A magneto-DNA nanoparticle system for rapid detection and phenotyping of bacteria. *Nat. Nanotechnol.* **8**, 369–375 (2013).
16. Gee, M. S. *et al.* Point of care assessment of melanoma tumor signaling and metastatic burden from µNMR analysis of tumor fine needle aspirates and peripheral blood. *Nanomedicine Nanotechnol. Biol. Med.* **13**, 821–828 (2017).
17. Lei, K.-M., Mak, P.-I., Law, M.-K. & Martins, R. P. CMOS biosensors for in vitro diagnosis – transducing mechanisms and applications. *Lab. Chip* **16**, 3664–3681 (2016).
18. Ha, D., Paulsen, J., Sun, N., Song, Y.-Q. & Ham, D. Scalable NMR spectroscopy with semiconductor chips. *Proc. Natl. Acad. Sci.* **111**, 11955–11960 (2014).
